# Changes in the Deceased-Donor Trend in Korea: Establishment of Regional Trauma Centers and KODA

**DOI:** 10.3390/jcm11051239

**Published:** 2022-02-24

**Authors:** Jeong-Moo Lee

**Affiliations:** Department of Surgery, Seoul National University College of Medicine, Daehak-ro 101, Chongno-gu, Seoul 03080, Korea; lulu5050@naver.com

**Keywords:** deceased donor, liver transplantation, regional trauma center, independent organ-procurement organization

## Abstract

South Korea utilizes living-donor liver transplantation to overcome a shortage of deceased donors. After the Korea Organ Donation Agency (KODA) was established, many hospitals were selected to be donor-managing hospital. A nationwide regional trauma center project was carried out separately in 2015. This study aims to analyze how the trend of deceased-donor recruitment and donation has changed based on policy factors such as independent organ-procurement organization (IOPO) activities and establishing regional trauma centers. KONOS data from 2010–2019 were used to analyze the impacts of these policy changes. The results showed that 62 centers had 4395 deceased donors, and 3863 recipients underwent deceased-donor liver transplantation. The two most common causes of donor death are cerebrovascular events and head trauma. When the rate of deceased donors was analyzed by the early period (2010–2014) and the late period (2015–2019), 53 non-trauma centers went from an average of 29.3 cases to 31.0 cases (6.2% increase) annually. Nine regional trauma centers showed a statistically significant increase from an average of 39.8 cases to 70.3 cases (75.9% increase) annually. Based on these policies, he locations where deceased donors are identified are changing. It is necessary to communicate with regional trauma center staff to recruit more deceased donors.

## 1. Introduction

Liver transplantation has been continuously developed in South Korea since it first began in 1988. The transplant centers in South Korea have plentiful experience in living-donor liver transplantation (LDLT) and have conducted the largest number of pure laparoscopic donor hepatectomies for liver transplantation worldwide. The reason for the rapid development of LDLT in South Korea is that it has been selected as an alternative to overcome the shortage of deceased donors. According to the Korean Network for Organ Sharing (KONOS) annual report in 2019, the rate of deceased-donor organ transplantation is still very low, with only 450 deceased donors in 2019, causing South Korea to be classified as a country in which deceased organ transplantation is not active [1]. Statistically, the number of donors per million population (PMP) is only 8.68 in Korea, as compared to 49.6 PMP in Spain.

In 2019, 40,253 patients waiting for organ transplantation were registered in Korea, an increase of 8.4% compared to the previous year. The wait for kidney transplantation is 3 years and 4 months on average, and that for liver transplantation is 2 years and 6 months. The number of patients who die while waiting for a transplant is 2136 per year.

Governments worldwide have attempted to solve the organ shortage problem. As a result, some countries, such as Spain, have secured enough deceased donors to treat their own population. When transplantations first began in the United States, its situation was similar to South Korea’s. However, a cooperative system between organ transplant centers and organ-procurement organizations was established in 1986. This led to the organ donation movement and the creation of new policies to increase organ utilization. Various attempts have been made to increase organ utilization, such as using marginal grafts, expanded-criteria donors, and donation after cardiac death [2,3,4,5,6].

In 2003, a specialized institution for determining brain death in hospitals was operated in the form of a hospital-based organ-procurement organization (HOPO). With the establishment of KODA as an independent organ-procurement organization (IOPO), a large number of donor-managing hospitals were selected as part of the policy, and deceased-donor management began at each site. As of 2021, 70 deceased-donor-managing institutions and 36 HOPOs are in operation to increase deceased-donor recruitment. In Korea, KONOS is responsible for approval of organ procurement from the deceased donors, and HOPO and IOPO conduct organ procurement (Figure S1).

Regional trauma centers were also established to intensively gather patients with severe trauma [7,8]. According to the 2019 KONOS Annual Report, 25% of deceased donors donate after trauma-related death. Until the regional trauma center project was established, patients with severe trauma were treated at nearby large-volume hospitals and hospitals without transplant centers or that had no education on organ transplantation [9,10]. Therefore, people who could be classified as potentially brain-dead usually died without making a donation or were discharged hopelessly after obtaining a Physician Orders for Life-Sustaining Treatment (POLST) form.

In the regional trauma center project, 17 centers were opened sequentially in each region, and the nationwide regional trauma center project was implemented in earnest in 2015 in South Korea. In the event of severe trauma, the Emergency Medical Service cooperated with each regional trauma center to transport patients, and potentially brain-dead donors were gradually gathered at the regional trauma center. In Korea, which still relies on living liver transplantation due to the lack of liver donors, intensive education on regional trauma centers and KODA’s efforts regarding organ transplantation should be conducted to increase the number of potential deceased donors.

The above-mentioned major national policies are expected to change the trend of discovering deceased donors in South Korea. However, no research has been conducted on the correlation between national policy and deceased donor recruitment so far.

This study aims to analyze how the trend of deceased-donor donations has changed according to national policy factors such as IOPO activities and the establishment of a regional trauma center in South Korea.

## 2. Materials and Methods

Among the KONOS data from 2010 to 2019, deceased donors were discovered and managed by hospitals and analyzed in relation to the establishment of regional trauma centers and the activities of IOPO. A deceased-donor-managing hospital is defined as a hospital that manages liver donation through deceased donors. The recipient hospital is defined as the hospital that conducted the liver transplantation.

### Statistical Analysis

The results are expressed as means ± standard deviation or as numbers and percentages. Continuous variables were compared using Student’s t-test, one-way ANOVA, and Kruskal–Wallis test; categorical variables were analyzed using the Chi-square test or Fisher’s exact test. A paired t-test was used to compare changes in the number of deceased donors in trauma and non-trauma centers. Statistical significance was defined as a two-tailed *p*-value < 0.05. All statistical analyses were performed using SPSS software (version 23.0; SPSS Inc., Chicago, IL, USA).

## 3. Results

From 2010 to 2019, a total of 62 centers had 4395 deceased donors and 3863 recipients underwent deceased-donor liver transplantation. The total number of deceased liver donors has increased in the past decade, but has been declining for the last five years. Since the establishment of IOPO, the number of donors at high-volume transplantation centers in Seoul has decreased overall, and they have been distributed to designated donor-managing hospitals. In the metropolitan area, the number of deceased donors in the largest five hospitals decreased from 612 to 392 (11.5%), while the number of deceased cases newly transferred to the regional trauma center increased from 356 to 628 (11.9%).

Table 1 shows the causes of death of the deceased donors. Cerebrovascular events were the most common cause of death, while intracranial hemorrhage, hypoxic brain damage, and traumatic brain injury-related death ranked second, third, and fourth, respectively; thus, head-trauma-related death, accounting for 26.1% of deceased donors, was the second most common cause. Whether cases classified as intracranial hemorrhage or hypoxic brain damage were associated with trauma could not be determined from the KONOS data alone. In addition, as each cause of death was duplicated in the KONOS data, there were more causes of death than the actual number of donors.

**Figure 1:**
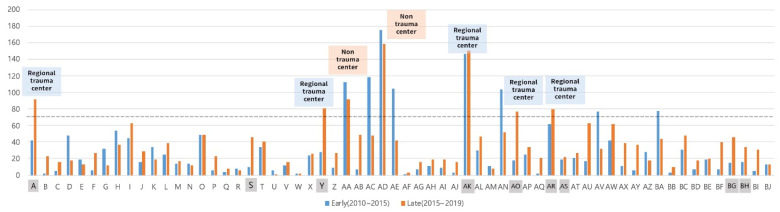
Comparison of recruitment numbers of deceased donors from regional trauma and non-trauma centers.

Using 2015 as the basis, when the regional trauma center began to be active, we divided the period from 2010 to 2019 into two parts to compare the total number of deceased donors by hospital (Figure 1). During the early period (2010–1014), the number of deceased donors from hospitals with high liver transplantation activity located in Seoul accounted for 32% of the total, but after the establishment of the IOPO and regional trauma centers the number of deceased donors in hospitals with high liver transplantation activity decreased significantly in the late period (2015–2019). However, the number of deceased donors in hospitals designated as regional trauma centers has begun to increase.

Except for the AD hospital (Figure 1), where the number of donors continued to be high regardless of policy changes, the number of deceased donors decreased in all high-activity transplant centers related to religious groups. AK hospital, where the trauma and transplant centers had been set up before 2015, had a large number of deceased donors without significant changes during the two periods. There were seven hospitals with more than 70 deceased donors per year for the last five years, of which five were regional trauma centers and only two were non-trauma centers.

**Figure 2:**
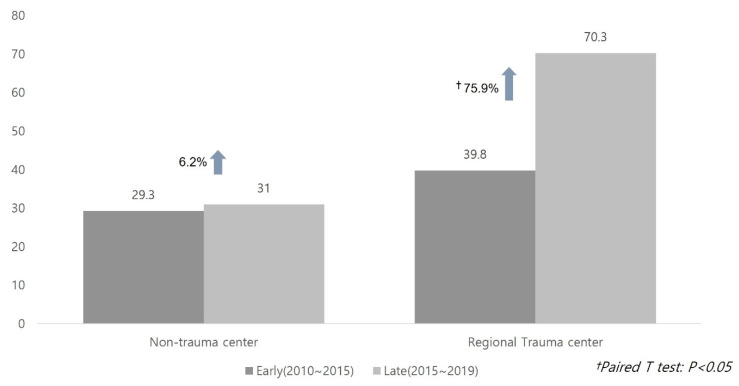
Changes in recruitment of deceased donors at regional trauma centers and non-regional trauma centers.

When analyzed by comparing trauma centers and non-traumatic centers, 53 non-trauma centers went from an average of 29.3 cases to 31.0 cases (6.2% increase). On the other hand, nine regional trauma centers showed a statistically significant increase from an average of 39.8 cases to 70.3 cases (75.9% increase) in the paired t-test (*p* < 0.05). In particular, the largest increase was seen in hospitals with regional trauma centers and transplant centers (Figure 2).

## 4. Discussion

The number of deceased donors in Korea is still low. In particular, Korea has a very large number of patients awaiting liver transplants due to the hepatitis B virus epidemic [11,12]. Although various alternative solutions such as ABO-incompatible liver transplantation have been introduced [13,14], it is still difficult to solve the fundamental problems of organ shortage, such as prohibiting donations to minors, and active recruitment of brain-dead donors may be the answer.

Since establishing the IOPO, there have been various efforts to discover deceased donors, but the annual changes in numbers of deceased donors has been insignificant, so it is difficult to see any significant results so far. Although it is a separate program, potential deceased donors have been coming to regional trauma centers since the mid-2010s because of the 2015 government policy. Since potential deceased donors are highly likely to be concentrated at regional trauma centers, the active promotion of organ donation programs should be discussed.

AK hospital, which accounts for the largest portion of donors, has not shown a significant difference between the early and late periods, but it was set up before 2015 and is a hospital with high levels of severe trauma and transplantation activity. The HOPO, which discovered most deceased donors in the early period, was related to a Catholic foundation due to the nature of the hospital, and many deceased donors were identified for external reasons even though it is not a regional trauma center. Excluding this center, the number of deceased donors in high-volume transplant centers decreased significantly. The primary reason for the dispersion effect is the IOPO policy, as well as the fact that most of the patients who donated were concentrated in regional trauma centers. A transplant center was established so that active HOPO-level brain-dead donor identification is active. The fact that hospitals with regional trauma centers and organ transplant centers show remarkable results proves the effectiveness of this approach.

However, regional trauma centers do not have status as donor-managing hospitals. Therefore, more deceased donors can be identified by collaborating with nearby hospitals with organ transplantation centers, securing the resident KODA staff, or acquiring the status of a donor-managing hospital. In addition, it is recommended that there be a mandatory organ-procurement program manager at the regional trauma center that can intensively manage potential deceased donors.

Liver transplantation and multiple trauma have many similarities in that they both require large amounts of blood transfusions, require advanced surgical skills, and require ICU care and anesthesia skills for critically ill patients. Rather than operating two independent centers, a hospital with an active organ transplant center will need a network that actively attracts trauma patients or collaborates with a regional trauma center. In the long run, it would be helpful to develop both types of centers (trauma and transplantation) at trauma centers to attract referrals from nearby hospitals with active organ transplants or to set up organ transplants. This could increase the possibility of attracting more donors in the future and reduce transfers between hospitals, thereby reducing the cold ischemic time and improving the long-term outcomes of liver transplantation.

One limitation of this study is that it only included hospitals that actually donated, meaning it is difficult to identify hospitals that found the deceased donor for the first time. Information about the identified hospitals was not included, even in the KONOS database.

Another limitation is that it is difficult to objectively compare the extent to which establishing trauma centers and the two independent factors of IOPO each influenced the actual deceased-donor donation trend. Although the establishment of IOPO and the regional trauma centers are completely separate projects, it is clear that they have had an impact on deceased-donor recruitment. However, since the state implemented each policy independently, it is difficult to identify their individual impacts. However, this study objectively showed that the deceased-donor recruitment trends can change depending on the direction of national policy. The significance of this study is that it provides a reference for policies aimed at increasing the efficiency of deceased-donor recruitment.

With the development of medical technology, the number of donors deceased due to disease may gradually decrease, although the ratio of trauma-related donations caused by increased leisure time or sports is likely to gradually increase. Many donor-managing hospitals were created after the IOPO was established, and among them regional trauma centers have made a strong leap forward. Unfortunately, although more than 80% of liver transplants are performed in Seoul, the city has no regional trauma center, so many patients donate from the surrounding area, which inevitably increases organ transfer times. If a regional or national trauma center is established in Seoul, many potential donors will be less likely to die without being told about the possibility of donation at a local hospital and without donating organs. The role of regional trauma centers in identifying deceased donors will become increasingly important, and active support and strategies are needed to facilitate this.

In conclusion, national policies and projects can change the organ donation trend. Organ-transplant-related hospitals, including KODA, need to cooperate with regional trauma centers in neighboring regions and comply with national policy changes. Transplant centers should make efforts to increase donations from multiple-trauma patients in South Korea, helping facilitate the identification of deceased donors and saving the lives of those waiting for transplantation.

## Figures and Tables

**Table 1:** Cause of death of deceased donors by year for the past 5 years.

Cause of Death ^†^	2015	2016	2017	2018	2019	Total
	501	573	515	449	450	2488
Stroke (infarct)	225 (44.9%)	229 (40.0%)	214 (41.6%)	160 (35.6%)	172 (38.2%)	1000 (40.2%)
Traumatic brain injury	130 (25.9%)	141 (24.6%)	117 (22.7%)	118 (26.3%)	127 (28.2%)	633 (25.4%)
Hypoxic brain damage	132 (26.3%)	191 (33.3%)	172 (33.4%)	165 (36.7%)	147 (32.7%)	807 (32.4%)
CNS tumor	3 (0.6%)	3 (0.5%)	3 (0.6%)	1 (0.2%)	2 (0.4%)	12 (0.5%)
Intracranial hemorrhage	348 (69.5%)	368 (64.2%)	328 (63.7%)	277 (61.7%)	292 (64.9%)	1613 (64.8%)
Asphyxia	82 (16.4%)	117 (20.4%)	95 (18.4%)	105 (23.4%)	91 (20.2%)	490 (19.7%)
Cardiovascular disease	29 (5.8%)	47 (8.2%)	46 (8.9%)	46 (10.2%)	32 (7.1%)	200 (8.0%)
Drowning	6 (1.2%)	3 (0.5%)	6 (1.2%)	4 (0.9%)	7 (1.6%)	26 (1.0%)
Epilepsy	7 (1.4%)	5 (0.9%)	4 (0.8%)	1 (0.2%)	4 (0.9%)	21 (0.8%)
Drug intoxication	2 (0.4%)	6 (1.0%)	2 (0.4%)	1 (0.2%)	2 (0.4%)	13 (0.5%)
Etc.	27 (5.4%)	27 (4.7%)	34 (6.6%)	15 (3.3%)	22 (4.9%)	125 (5.0%)

^†^ Each cause of death was recorded in the KONOS database, with duplicate values allowed.

## Data Availability

Data are available in a publicly accessible repository that does not issue DOIs (https://www.konos.go.kr/konosis/index.jsp, accessed on 11 November 2021).

## Supplementary Materials

The following supporting information can be downloaded at: www.mdpi.com/article/10.3390/jcm11051239/s1, Figure S1: Flowchart of potential deceased donor organ procurement in South Korea; KODA, Korea Organ Donation Agency; KONOS, Korean Network for Organ Sharing; IOPO, Independent Organ-Procurement Organization); HOPO, Hospital-based Organ-Procurement Organization.
