# Supplementary material for: Changes in the Deceased-Donor Trend in Korea: Establishment of Regional Trauma Centers and KODA
Source: J Clin Med. 2022 Feb 24;11(5):1239. doi: 10.3390/jcm11051239 (PMC8911019; doi:10.3390/jcm11051239)
Supplement: Supplementary file 1 [file jcm-11-01239-s001.zip › jcm-1575505-supplementary.pdf]

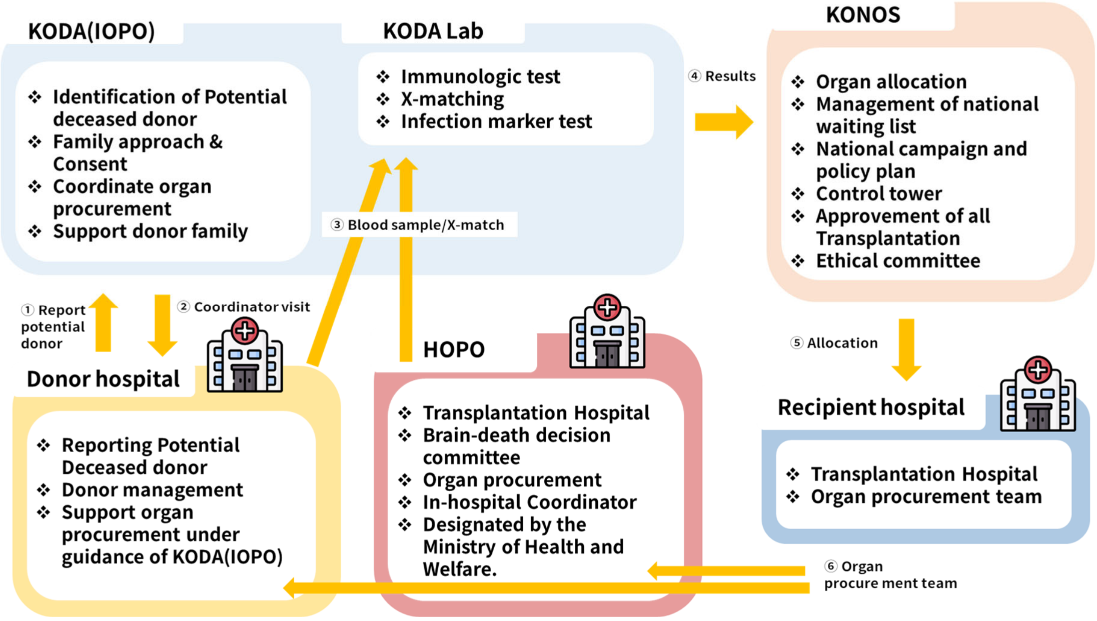

Figure S1. Flowchart of potential deceased donor organ procurement in South Korea; KODA, Korea Organ Donation Agency; KONOS, Korean Network for Organ Sharing; IOPO, Independent Organ-Procurement Organization); HOPO, Hospital-based Organ-Procurement Organization
